# Effect of the level of task abstraction on the transfer of knowledge from virtual environments in cognitive and motor tasks

**DOI:** 10.3389/fnbeh.2023.1162744

**Published:** 2023-04-18

**Authors:** Viviana del Rocío Hernández-Castañón, Arlem Aleida Castillo-Ávila, Verónica Reyes-Meza, Nadia Bianchi-Berthouze, Alberto L. Morán, Felipe Orihuela-Espina

**Affiliations:** ^1^Department of Computational Sciences, Instituto Nacional de Astrofísica, Óptica y Electrónica, Puebla, Mexico; ^2^Centro Tlaxcala de Biología de la Conducta, Universidad Autónoma de Tlaxcala, Tlaxcala, Mexico; ^3^UCL Interaction Centre, University College London, London, United Kingdom; ^4^Faculty of Sciences, Autonomous University of Baja California, Ensenada, Mexico; ^5^School of Computer Sciences, University of Birmingham, Birmingham, United Kingdom

**Keywords:** virtual environment, transfer of knowledge, task abstraction, electroencephalography, virtual training, real environment

## Abstract

**Introduction:**

Virtual environments are increasingly being used for training. It is not fully understood what elements of virtual environments have the most impact and how the virtual training is integrated by the brain on the sought-after skill transference to the real environment. In virtual training, we analyzed how the task level of abstraction modulates the brain activity and the subsequent ability to execute it in the real environment and how this learning generalizes to other tasks. The training of a task under a low level of abstraction should lead to a higher transfer of skills in similar tasks, but the generalization of learning would be compromised, whereas a higher level of abstraction facilitates generalization of learning to different tasks but compromising specific effectiveness.

**Methods:**

A total of 25 participants were trained and subsequently evaluated on a cognitive and a motor task following four training regimes, considering real vs. virtual training and low vs. high task abstraction. Performance scores, cognitive load, and electroencephalography signals were recorded. Transfer of knowledge was assessed by comparing performance scores in the virtual vs. real environment.

**Results:**

The performance to transfer the trained skills showed higher scores in the same task under low abstraction, but the ability to generalize the trained skills was manifested by higher scores under high level of abstraction in agreement with our hypothesis. Spatiotemporal analysis of the electroencephalography revealed higher initial demands of brain resources which decreased as skills were acquired.

**Discussion:**

Our results suggest that task abstraction during virtual training influences how skills are assimilated at the brain level and modulates its manifestation at the behavioral level. We expect this research to provide supporting evidence to improve the design of virtual training tasks.

## 1. Introduction

A virtual environment (VE) has been defined as a computer-generated environment used to simulate the real environment (RE) (Gupta et al., 2008). The development of VEs have increased in the last years in different areas, such as security (Passos et al., 2016), health (Kalyvioti and Mikropoulos, 2014), education (Popovici et al., 2005), rehabilitation (Orihuela-Espina et al., 2013) and entertainment (Macedonia, 2000), among others. VEs have gained popularity for task training because they provide a relaxed, non-threatening environment that allow users to learn from their mistakes without irreversible consequences (Bertram et al., 2015). In addition, these training environments help designers to incorporate feedback mechanisms for enhancing the user experience (Gupta et al., 2008).

Training in VEs relies on the assumption that acquired skills and/or knowledge can be transferred to the RE. Indeed, the training from VEs is useful, insofar as it is possible to transfer the acquired knowledge toward its counterpart in the RE (Bossard et al., 2008). This perspective has been named as *the transfer of knowledge*. Broadly speaking, transfer of knowledge is a process by which knowledge constructed in a particular context (source task) can be used in a different context (target task) after being mobilized, recombined, and/or adapted (Bossard et al., 2008). In using VEs for training, however, it is necessary to understand how the transfer occurs and what factors enable the mobilization of knowledge when a new knowledge is constructed by abstraction and the learner is confronted with variable situations. Many success cases have been reported of using VEs as training tools (Rouiller and Goldstein, 1993; Webber et al., 2001; Popovici et al., 2005; Yang et al., 2007; Kiper et al., 2014), yet some studies were unable to show any transfer (Bossard et al., 2008; Khan et al., 2015). This previous evidence supports the fact that the transfer of knowledge from VE to RE is possible, but, there are unresolved issues.

Studies have been conducted to evaluate the learning of tasks from VEs through its contents and training mechanisms (Todorov et al., 1997; Rose et al., 2000; Bossard et al., 2008; Girvan and Savage, 2019). In addition, the selection of educational content in the design of VEs remains an important but nontrivial step for successful training and learning tasks. On the transfer of knowledge, Subedi (2004) discussed that it is relatively easy to learn a task, and the transfer rate is usually high under the training of tasks that are procedural in nature which include the steps of the operation sequence, and the sequence of steps is repeated every time the task is performed. However, it is insufficient and unlikely to adapt such skills and knowledge when the learner is confronted with a new environment and changing conditions. From this perspective, in the learning task domain, abstraction has become an important research subject that helps to understand how knowledge is constructed and generalized.

Abstraction involves a structured process of initial concept formulation and generalization of ideas from concepts which lack a perceivable referent. Abstract concepts do not refer to physical objects that can be directly experienced by the senses (Kiefer and Harpaintner, 2020). Therefore, their semantic content is less obvious compared to concrete concepts and consequently harder to understand, process, acquire, and remember, which entail the act of generalizing something by taking out only important points from detailed characteristics of a problem. Hence, the construction of knowledge and its generalization imposes challenges in cognitive psychology, so that, future researches are addressed to systematically investigate experience-dependent plasticity of abstract concepts at the functional and neural levels as a function of training or expertise.

Research on learning of abstract and concrete concepts has a long history. Theoretical considerations are based on the view that abstract concepts require amodal symbolic (Mahon and Caramazza, 2009) or verbal representations (Paivio, 1986). Such views are also present in current theoretical considerations. Paivio, on his influential Dual Coding Theory (DCT) (Paivio, 1986), suggests ways in which the cortical representations of various tasks and contexts are activated via the connecting pathways, assuming that abstract concepts are stored in a verbal-symbolic code, while concrete concepts rely on both a visual imaginary and verbal-symbolic code. The basis of this theory has been essential to explain findings in early studies that have investigated differences in the processing between abstract and concrete concepts at a behavioral and neural level. Based on this theory, abstract concepts have been treated as a homogeneous conceptual category defined by a referent with a lack of unique physical features (Kiefer and Harpaintner, 2020). For instance, it has been suggested that concrete words are remembered better (Marschark and Paivio, 1977) and recognized faster than abstract words (James, 1975). On the other hand, the model of lexical processing developed by Alfonso Caramazza and colleagues (Caramazza and Hillis, 1991; Caramazza, 1997) points that sensory or motor information are transformed into a common amodal representation format, detached from modality-specific information (Kiefer and Harpaintner, 2020). The activation of sensory and motor systems during conceptual processing serves to ground “abstract” and “symbolic” representations in the rich sensory and motor content that mediates our physical interaction with the world (Mahon and Caramazza, 2008). Some works based on amodal theories are semantic network models (Quillian, 1969; Collins and Loftus, 1975) or connectionist network models (Caramazza et al., 1990; McClelland and Rogers, 2003; Rogers et al., 2004).

With the arrival of neuroimaging recordings, different patterns of neural activation supporting abstract and concrete concepts have been observed. Consistent with the DCT theory, neuroimaging techniques such as positron emission tomography (PET) or functional magnetic resonance imaging (fMRI) have revealed lateralized responses; in the left hemisphere in presence of abstract concepts and in the right hemisphere in presence of concrete concepts (Binder et al., 2005). The evidence is still growing. Some studies have shown left hemisphere activity for both concepts (Sabsevitz et al., 2005) with greater activity within language regions for abstract concepts and greater activity within the visual network for concrete concepts (Desai et al., 2011; Sakreida et al., 2013). Others studies with activity within the sensory-motor system for abstract and concrete concepts (Pexman et al., 2007), sometimes complemented by activation in the left hemisphere language regions (Desai et al., 2011) or greater right hemisphere activation in presence of abstract words (Kiehl et al., 1999). Electrophysiological activity detected by electroencephalography (EEG) signals has also reflected the existence of different physiological response of encoding and retrieving in abstract vs. concrete concepts (Welcome et al., 2011; Bechtold et al., 2019), elucidating that abstract elements may relate to more attention to internal processing of the task (Harmony et al., 1996) and concrete elements to reduce response time during elements association relative to abstracts elements (Bastiaansen et al., 2005). In the analysis of the brain regions modulated by task demands, these above studies have provided evidence of how abstract concepts can activate specific subregions in the brain and contribute to decision-making and mobilizing knowledge (O'reilly et al., 2002; Koechlin et al., 2003; Christoff et al., 2009; Dixon and Christoff, 2014). Beyond examination of the transient effect of the level of abstraction in cortical or subcortical regions in the brain, it remains necessary to analyze the representations and associations during learning as a process that occurs in response to the demand of tasks.

Analyzing these aspects could clarify the mechanisms and interventions to guide the knowledge acquisition at cognitive and behavioral level. To find these answers in this active topic of research, the EEG signal has been particularly useful in studying the cognitive functions of the brain (Ray and Cole, 1985; Halgren and Marinkovic, 1995; Mikropoulos, 2001), providing noninvasive access to the brain electrical activity with higher temporal resolution than other functional neuroimaging approaches.

From theory-driven approaches frequently used for investigating the knowledge linked to concrete and abstract concepts, this research aims to study the relationship between the level of task abstraction and the progress of observable performance, following a period of training in a set of two tasks (cognitive and motor) implemented on computer screens simulating virtual environments, elucidating whether the level of abstraction is, in part, responsible for the process of transfer of knowledge acquired from a VE to the RE. We hypothesized that the level of task abstraction in the VE modulates the cognitive integration of the training; either by allowing high performance in the RE execution, with limited generalization capacity, or by compromising specific performance according to a greater generalization. To support (or refute) such an approach, we contribute with new evidence about how the level of task abstraction impacts the transfer of knowledge from a VE to the RE. This research has implications in the HCI area, where it remains necessary of providing methodologies to neuroergonomically guide the development of VEs for training.

## 2. Materials and methods

### 2.1. Task definition and characterization of levels of task abstraction

**Figure 1:**
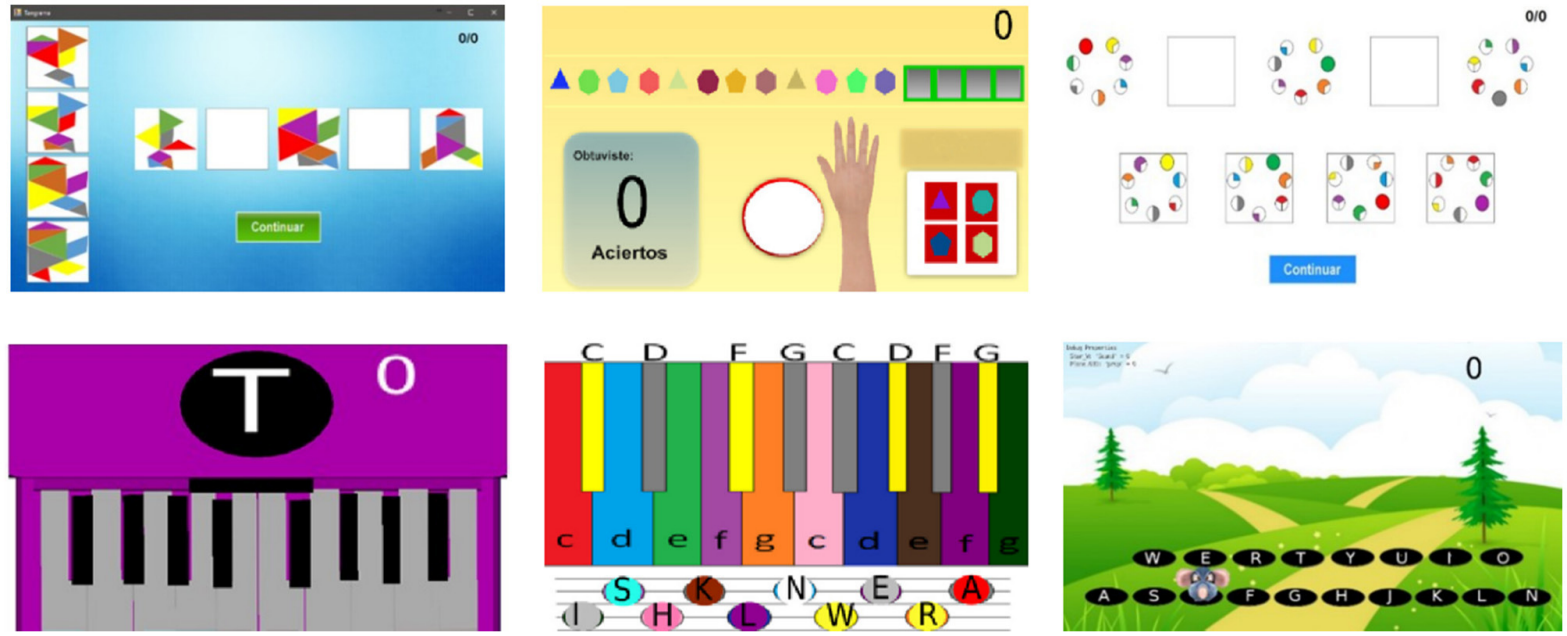
Tasks implemented in the virtual environments. **(Top)** Cognitive task, **(down)** Motor task. The middle level of abstraction **(middle)** was used for piloting and as a baseline reference for the low **(left)** and high **(right)** levels of abstraction but was not used during experimentation.

Aided by an expert psychologist (VRM), one cognitive task and one motor task were designed with three levels of abstraction to each of them (see Figure 1). The middle level of abstraction was used during the piloting of the experiment to validate the tasks implemented in the virtual environments, the training times, and verify the abstraction modulation (panel on the middle). The other two levels of abstraction, low and high, were used during experimentation. After piloting, elements of the middle level of abstraction were rescued to modulate the low and high levels of abstraction. For the cognitive task, the principle of a logical sequence was followed. The geometrical patterns that integrated the sequence, their rotations, as well as the colors were included in the task regardless of the level of abstraction. For the motor task, skills regarding fractionation of fingers contributing to visuospatial processing were evaluated. The challenge time remained equal across levels of abstraction.

The cognitive task, modulated into two levels of abstraction, consisted of solving logical sequences of geometrical shapes using the given information to find the two missing intermediate steps. The focus on the construction of logical sequences has been linked to the content perception according to the internal structure that has been represented by every logical sequence, either concrete objects or abstract elements. The low level of abstraction was based in the tangram game and consisted of a five-step sequence following a pattern of movements over seven geometric pieces with different colors and sizes to create a figure representing a concrete object at the end of the sequence (top-left). The high level of abstraction consisted of a five-step sequence following a pattern of rotations in circles with seven characteristics represented through colors (top-right). To modulate the level of abstraction, the task under the low abstraction followed a pattern of movements in the geometric shapes which carried to represent a concrete object. On other hand, the task under the high abstraction, representation of a concrete object, was abstracted by only dropping off a pattern of movements and geometric shapes. Importantly, regardless of the level of abstraction, the task complexity remained constant; the task space consisted of variations in two parameters: number of geometric shapes and spins. We had seven geometric shapes and four possible rotations (90° rotations for each geometrical shape): 7 × 4 for a total of 28 possible combinations.

The motor task, modulated into two levels of abstraction involved fractionated finger movements following a random letter sequence. This task aimed to master the motor skills contributing to visuospatial processing either by typing letters from the constructed representation of a piano for the low level of abstraction or by detecting motions on the screen and react and tap for the high level of abstraction. The low level of abstraction of the task consisted of a piano keyboard representation on the screen, and by typing on the computer keyboard, a sequence of letters appeared one by one on the screen at every instant of time (bottom-left). The task did not demand the association between the keyboard keys and real piano keys. The high level of abstraction was inspired in the Whack-A-Mole game and consisted of hitting a target by typing on the computer keyboard the letter where the target appeared (bottom-right). Again, task difficulty was kept constant across abstraction levels; the task space consisted of 18 letters distributed equal to the number of keyboard keys and the sequence of keystrokes was randomized.

### 2.2. Virtual and real training

**Figure 2:**
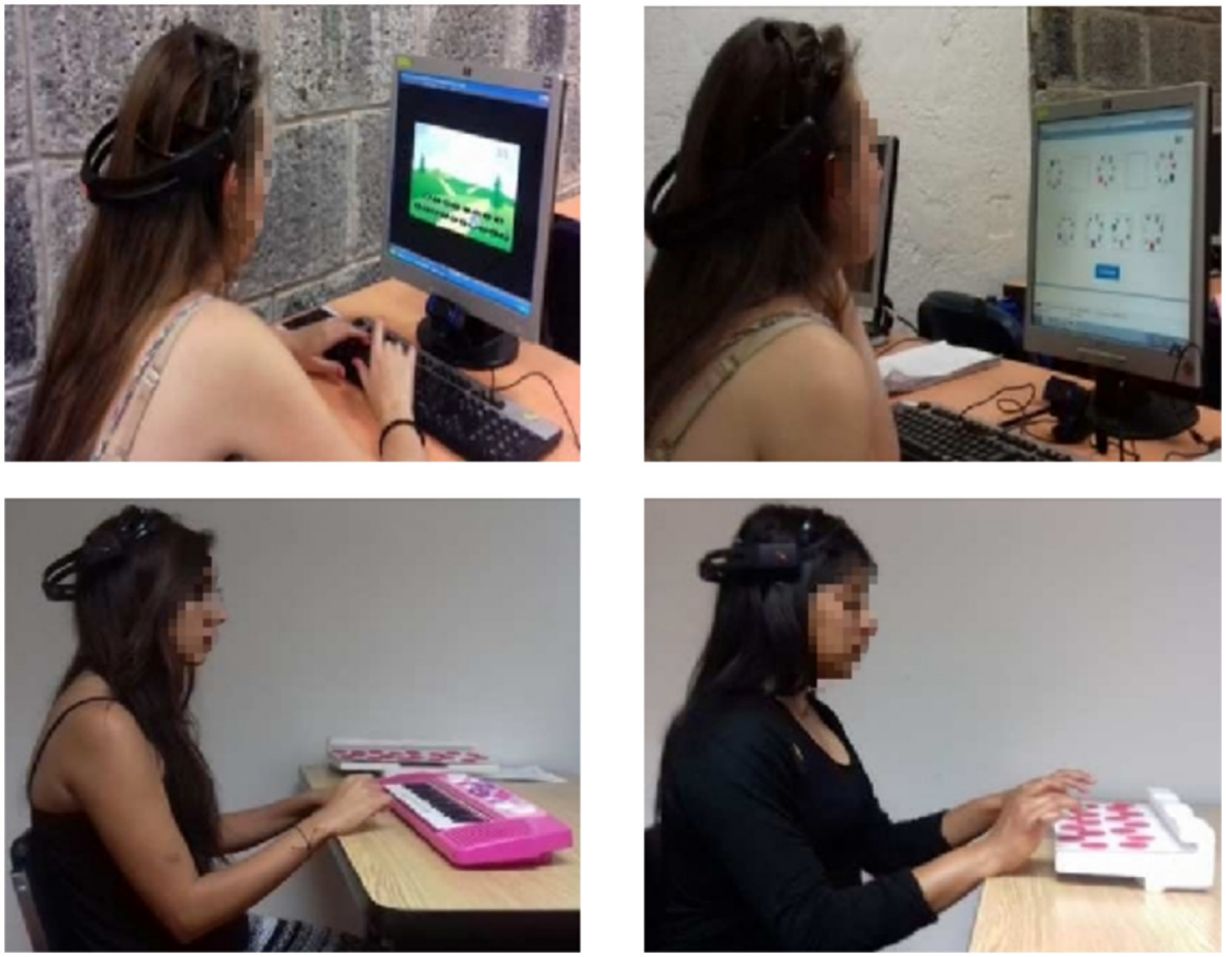
Training in the virtual **(top)** and real **(bottom)** environments.

Virtual training occurs in a virtual environment controlled with regular input interfaces to a desktop computer. Real training occurs in a physical environment where interaction occurs directly with the depicted instruments and devices. The implementation of these tasks under the different levels of abstraction was presented in a virtual environment rendered on a screen and the interaction with the environment occurs through the computer input devices, e.g., a computer keyboard (see Figure 2). On the other hand, in the real training, the tasks were presented in the physical environment, and interaction occurs by direct physical manipulation of the physical objects involved, e.g., an (electronic) musical keyboard. Supported by physical components, real scenarios were constructed of these two tasks with their appropriate abstract items. To carry out equivalent trainings, the virtual environments (VEs) were implemented as closely resembling the real scenarios of these tasks as possible.

### 2.3. Experimental design and setup

Twenty five participants, 20 female and 5 male, between 18 and 23 years of age recruited from BLINDED FOR REVIEW, participated in the study. The experiment was explained to the participants, and they gave their informed consent before participation. Following consent to participate, using simple randomization, subjects were randomly assigned to real (*RT*) or virtual (*VT*) training, and either high (*HA*) or low (*LA*) level of task abstraction training. This was a between-subjects bi-factor design of four training schemes or treatments (*T*); *RT*−*HA* (*N* = 6), *RT*−*LA* (*N* = 6), *VT*−*HA* (*N* = 6), and *VT*−*LA* (*N* = 7). Data were recorded longitudinally (pre-post). The response variable was the transfer of knowledge of the participants at the completion of the training, and the level of task abstraction was the factor whose effect on the transfer of knowledge level has been investigated.

The inclusion criteria for the participants were age range, ability to understand instructions, no record of psychological or psychiatric disorders, and no previous serious training in VEs (in any task, but they may be otherwise regular computer gamers) or piano playing. Furthermore, participants were selected with same profile and study level to avoid potential bias in abstraction skills (e.g., stronger mathematical training). We made no gender distinction. Exclusion criteria only accounted for participation in piloting. Regarding the elimination criteria, participants who did not complete the trial were discarded.

**Figure 3:**
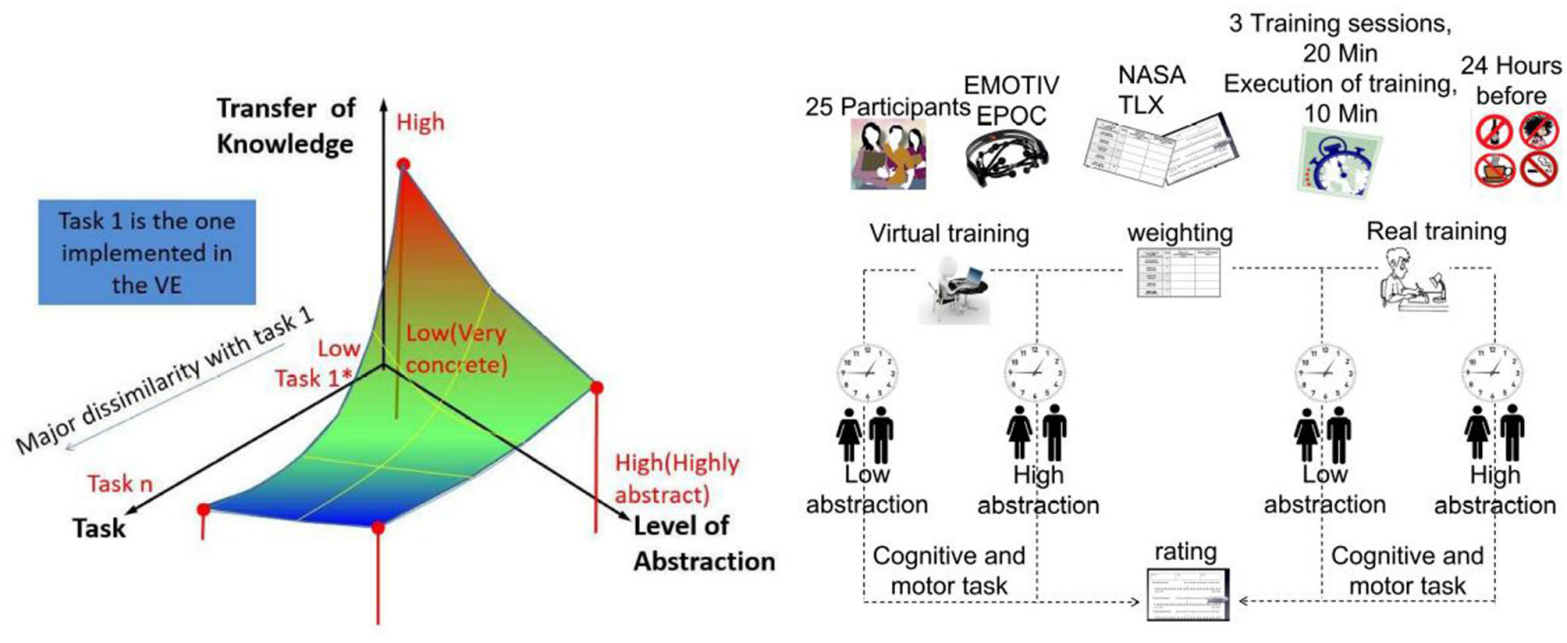
**(Left)** Schematic representation of the hypothesis: when the level of abstraction in a task is low, a high transfer of knowledge is reached but it compromises the mobilization of knowledge toward other task. When the level of abstraction in a task is high, the transfer of knowledge occurs in a moderate way but the skills to face other task are improved. **(Right)** Representation of the experimental design: on the top, cohort size, assessment tools, and procedure are indicated. On the lower part, the training path followed by the groups is illustrated.

For experimental sessions, the participants were asked to refrain from drinking alcohol, smoking, and drinking coffee during the 24 h period preceding the experiments and to keep a regular schedule and go to sleep at their usual bedtime the night before the experiments. Figure 3 schematically depicts the hypothesis and experimental design. All groups received the same amount of training; one 20 min session for 3 consecutive days. On the fourth session (post-training), regardless of the training treatment received, the subjects executed the trained tasks in the real scenario for 10 min, and to verify the possible associations related to the abstraction of the trained task, they performed for 10 more minutes the task with the opposite level of abstraction to the trained. The execution of the task was fixed in 10 min, both for the same abstraction and the opposite abstraction, taken as a necessary action from the exhausting training that participants expressed had performed. Nevertheless, this determination was not an impediment to evaluate the training using the corresponding criteria. Blinding participants to treatment was not possible as the participants had to explicitly carry out the training.

A pilot was carried out using the middle level of abstraction of the cognitive and motor task, to ensure the competences of the participants along the training sessions and consequently to modulate parameters in the task and training sessions. The intrinsic elements of the task as well as the number of sessions and the training times were adjusted by the piloting. Volunteers who participated in the pilot were not eligible for the main study. All participants had the same time for training, however consequently, they performed a different number of sequences, which later was normalized in the range of 0–100 for each of them (see Section 2.4).

### 2.4. Performance assessment

Whether virtual or real training and high or low abstraction of the cognitive and motor task, the performance of each participant was assessed using hit scores. For the cognitive task, a hit was scored when two missing patterns were correctly identified in the intermediate boxes of every logical sequence trial. The time limit was fixed for the entire session. For the motor task, in the piano game, every hit was scored by typing the correct letter shown one by one on the screen, and in the Whack-A-Mole game, by knocking the mole on the head as it appeared. The time limit for responding to the target, either the letter or the mole, was fixed considering the reaction time as the challenge when performing this task. The recording of the scores was carried out along the four longitudinal sessions (3x training according to treatment + 1 final assessment on the RE). Scores obtained from the first session day were considered as the baseline of the participant. The next two sessions in subsequent days covered the training period. Finally, the fourth session was divided into two stages: in the first stage, the trained task was executed in the RE by the participant and in the second part, the opposite level of abstraction to the trained task was executed in the RE by the participant. To calculate the performance of each participant, the number of successes (correct exercises solved) was divided by the total number of exercises done by session. Even though the time assigned to each treatment was the same, each participant solved a different number of exercises. Therefore, the values obtained from the division were normalized in the range of 0–100 in all cases.

### 2.5. Mental workload assessment

The perceived workload by the participants was assessed using the NASA TLX questionnaire (Aeronautics and Administration, 2018). The evaluated experimental factors were type of training (virtual vs. real), task nature (cognitive vs. motor), and level of abstraction (low vs. high). This questionnaire was applied in two steps: weighting and rating (see Figure 3). Both parts were applied on the first day of training and when executing in RE the trained task. During the first part (pre-session), subjects evaluated the contribution of each factor to the workload of the task. The factors are six scales that were combined pair-wise. The weights provide diagnostic information about the nature of the workload imposed by the task. During the rating part (post-session), the subjects assigned numerical rating in each scale divided into 20 equal intervals (e.g., low/high). This rating assigned for each scale reflect the magnitude of the task. The overall workload score for each subject was computed by multiplying each rating by the weight given to that factor by that subject. The sum of the weighted ratings for the task was divided by 15 (the sum of the weights).

### 2.6. Electroencephalography: recording, processing, and analysis

**Figure 4:**
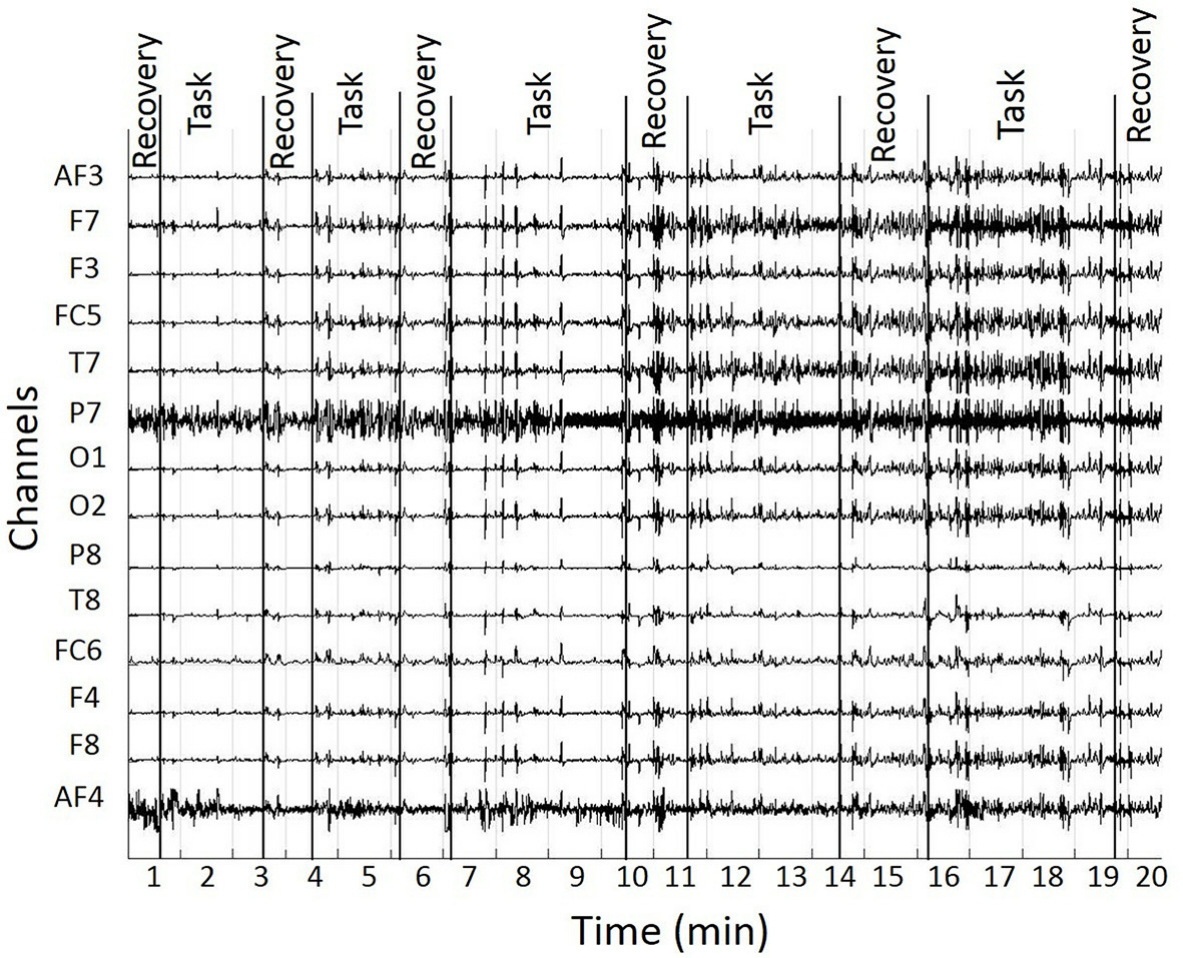
Raw EEG signal from one participant. It was segmented in epochs of recovery and performance during the task training.

EEG signals were registered on the first day of training, and later when executing the trained task in the RE. EEG was recorded using an Emotiv EPOC^®^ kit (Emotiv, San Francisco, USA) at 14 channels from positions AF3, F7, F3, FC5, T7, P7, O1, O2, P8, T8, FC6, F4, F8, and AF4 of the 10/20 system (Jurcak et al., 2007). Data were recorded at 128 Hz. Figure 4 shows an exemplary raw signal from a participant.

**Figure 5:**
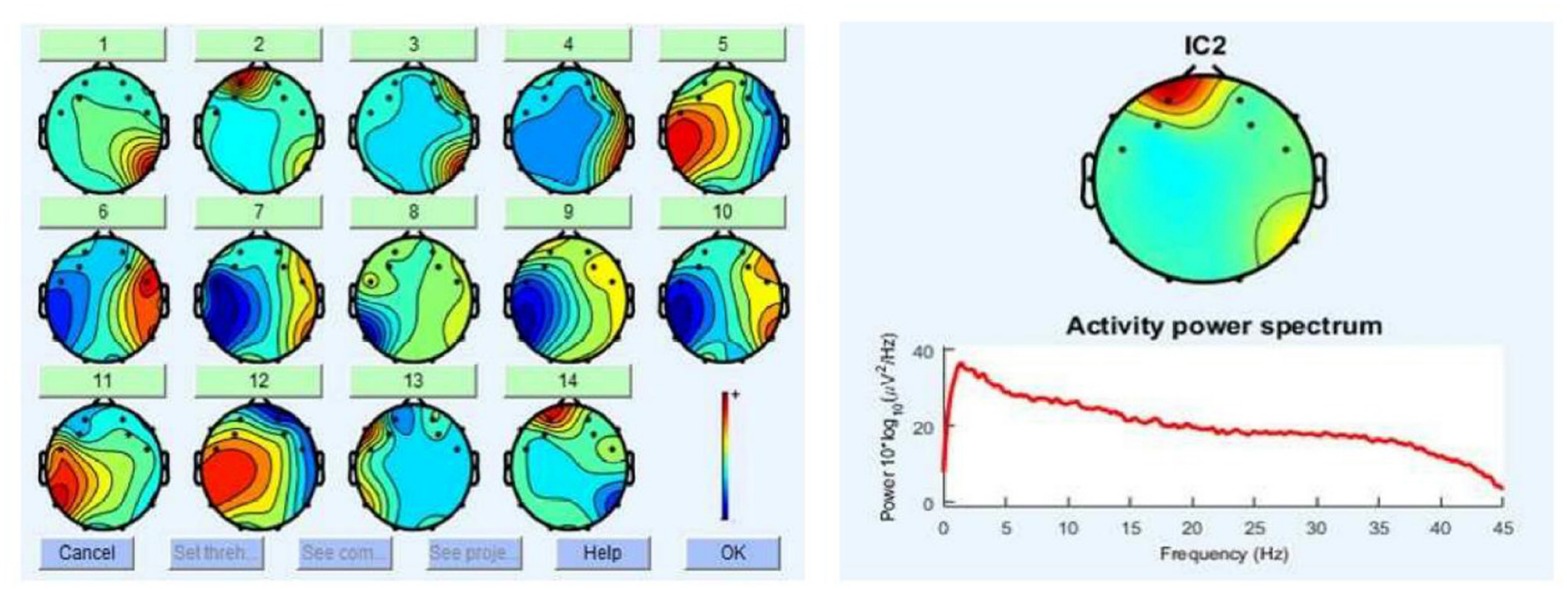
**(Left)** Topographic maps using independent components analysis (ICA). The spatial topography of each component represents its distribution in the different electrodes. This contribution is shown in scale of colors that reflect the energy in each brain area. **(Right)** Independent component detected as a potential artifact by the automatic algorithms, ADJUST Mognon et al. (2011) implemented in EEGLAB. Top is shown the spectral contribution of component in the brain topography and down is shown the associated power spectrum.

Preprocessing was applied to EEG signals to reduce the noise of the signal of interest. A band pass filter between 2 Hz and 50 Hz was applied that should comfortably cover the interval generally associated with cognitive activity (Lan et al., 2005), while excluding the line noise. Blinking, eye movements, and generic discontinuities were alleviated with ICA (Independent Component Analysis) using EEGLAB toolbox^®^. The automatic ADJUST algorithm (Mognon et al., 2011) identified the independent components affected by some artifact by combining stereotyped artifact-specific spatial and temporal features. Affected components were removed from the data. In Figure 5, left, the components detected by ICA are shown, and in Figure 5, right, one of the components, for which its spatial and spectral characteristics is related to a typical blink (Jung et al., 2000), is shown.

EEG analysis consisted of the segmentation of continuous periods of similar activity as identified by an automatic constraint clustering algorithm over time, frequency, and topographical space (del Roćıo Hernández-Castañón, 2017). This analysis was implemented in MATLAB (R2017b, Mathworks, USA), but in brief (del Roćıo Hernández-Castañón, 2017):

1. Time, Frequency, and Topography (TFT) projections of the EEG were generated using the short-time Fourier transform (STFT).
2. The spatial activity corresponding to channels across time and frequency was rearranged in preparation for clustering.
3. Segmentation of TFT-epochs was obtained from neuronal activity signature using the constrained clustering technique. Hierarchical cluster analysis (HCA) was chosen because of the quality of clustering it generates (Jain and Dubes, 1988). The constraints were defined according to the knowledge of the phenomenon which was based on the similarity and adjacency principles of the cognitive sub-processes.

The analysis of brain activity was elaborated over the identification of automatically segmented TFT-epochs assumed to represent different cognitive subprocesses. The assessment of brain status was done using the entropy function from Moddemeijer's library.

### 2.7. Statistical analysis

Statistical analysis was carried out to establish whether there was a significant effect on the transfer of knowledge, as a consequence of the level of task abstraction or the type of training. We have performed separate analyses, by type of training (virtual vs. real, regardless the abstraction level) and by level of abstraction (low vs. high, regardless the training). In both cases, a fixed effects general linear model was used.

Power analysis carried out before experimental data collection aimed for customary 95% statistical power with 5% significance level and assumed effect size (Cohen's *d* = 0.4). The required sample size for such power was *N* = 27 per treatment. By the end of the pre-scheduled recruitment period, we only succeeded to enroll 40 participants meeting the inclusion criteria, without payment for participation to avoid bias (Camerer and Hogarth, 1999). Recruitment effectiveness was low due to a variety of factors; absence of monetary incentive coupled to limited advertisement and an unanticipated short recruiting period due to our overestimation of our capacity to recruit. However, changing or enlarging the pre-established recruitment period is a discouraged practice by the CONSORT standard (Moher et al., 2012). Hence, unfortunately, we were unable to reach the targeted sample size. Moreover, some participants did not complete their randomly assigned treatments over the four sessions due to the high time demands from the volunteers, leading to a larger than anticipated drop out rate. In total, 15 participants were excluded for further analysis. Consequently, a second posterior power analysis was further carried out to estimate the residual statistical power with the acquired sample, and with *N* = 25, the margin of error was evaluated at 19% from the 95% statistical power. This circumstance does not invalidate the results since we are compliant with the standard as stated, but reduces the statistical power of the study as reported.

One-tailed Mann–Whitney U analysis was carried out over the scores to evaluate the performance of the participants by type of training and level of abstraction, as well as, to compare the assessment of the mental workload measured by the NASA-TLX in the same experimental factors, and to compare the differences in the electrophysiological response (defined by the entropy) of the brain recorded by the EEG signal.

Statistical analysis was conducted using SPSS version 24.0 (IBM Corporation, USA).

## 3. Results

Table 1 summarizes the demographic data from the four training treatments: *RT*−*HA*, *RT*−*LA*, *VT*−*HA*, and *VT*−*LA*, as well as the baseline score in the cognitive and motor task per group.

**Table 1:** Cohort demographics.

		***RT*−*HA***	***RT*−*LA***	***VT*−*HA***	***VT*−*LA***
		**(*n* = 6)**	**(*n* = 6)**	**(*n* = 6)**	**(*n* = 7)**
Male/Female		1/5	2/4	0/6	2/5
Age (mean)		18.7 ± 0.8	18.8 ± 0.8	19.2 ± 1.9	19.4 ± 1.5
Baseline score	Cognitive	24.6 ± 15.6	45.4 ± 22.0	14.3 ± 12.5	16.1 ± 23.3
		Motor	92.2 ± 3.2	84.6 ± 5.3	69.2 ± 7.1	65.5 ± 10.1

Count is indicated for sex. Mean and standard deviation is indicated for age and score.

### 3.1. Analysis of performance

The performance of the received virtual (intervention) vs. real (control) groups as well as the effect of the level of abstraction in the transfer and mobilization of knowledge were compared using scores recorded in each session of training, when executing the trained task and when executing a task with the opposite level of abstraction of the trained task. The analysis was made for both the cognitive and motor tasks.

#### 3.1.1. Assessment of the transfer of knowledge by type of training

**Figure 6:**
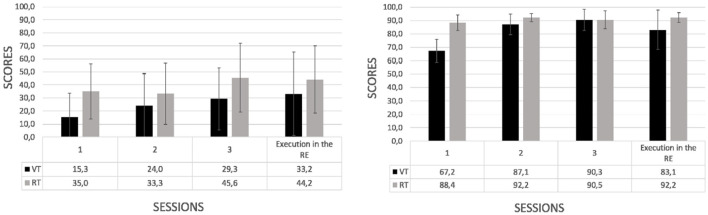
Performance scores (mean and standard deviations) across longitudinal sessions by type of training of the cognitive **(left)** and motor **(right)** task. The effect on the behavioral performance due to the virtual (VT) vs. real (RT) training was compared.

Figure 6 illustrates the results on the transfer of knowledge as assessed by the normalized score structured by the experimental factors that contrast type of training: virtual (intervention) vs. real (control). According to this factor, virtual training over the real training led to higher scores when comparing the performance of the participants during their first session of training against their performance during the post-training (execution in the RE the trained task). This occurred both in the cognitive (left) and motor (right) tasks, although differences did not reach significance (performance scores in the cognitive task, *VT*:17.9 ± 39.6 vs. *RT*:9.2 ± 16.6, *U* = 65, *p* = 0.25; performance scores in the motor task, *VT*:15.9 ± 17.6 vs. *RT*:3.9 ± 4.5, *U* = 25, *p* < 0.05). Even though participants trained in the real environment started the training with higher scores over participants trained in the virtual training, the performance along the training sessions and when executing the trained task was better in the virtual training. Therefore, a better achieved performance of the treatment was not preceded by a higher score in the first session training. Concerning the transfer of knowledge, the evidence suggests that the learning of the task was better achieved in the VT than the RT with the improvement scores from the first (session 1) to the fourth sessions (execution in the RE) of both the cognitive and motor tasks.

#### 3.1.2. Assessment of the mobilization of knowledge by level of abstraction

**Figure 7:**
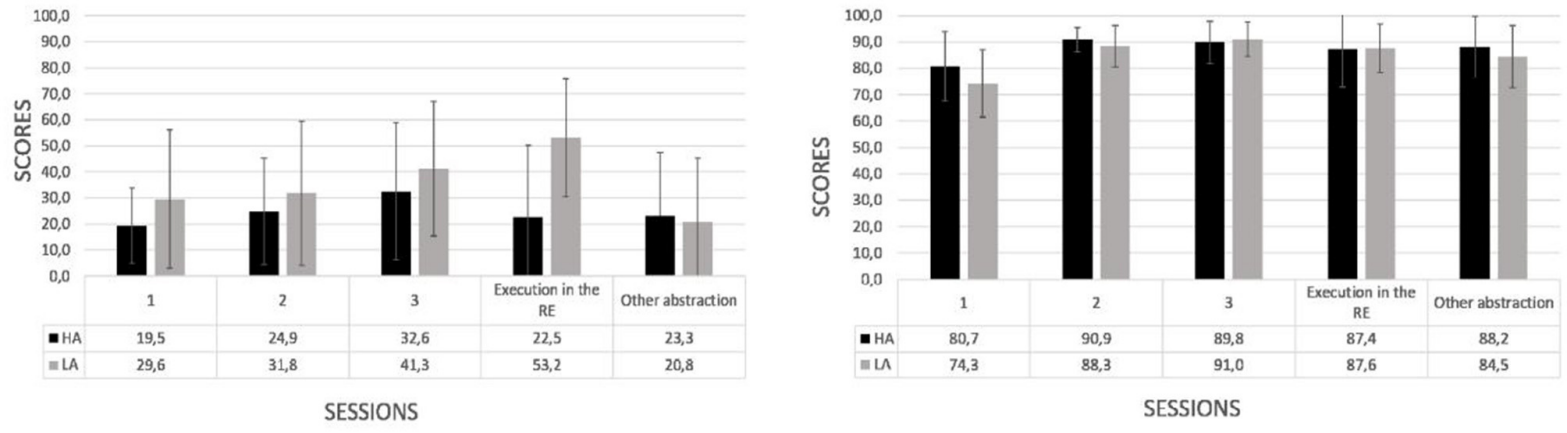
Performance scores (mean and standard deviations) across longitudinal sessions by level of abstraction of the cognitive **(left)** and motor **(right)** tasks. The corresponding effect of the high (HA) vs. low (LA) level of abstraction on the mobilization of knowledge was compared.

With regard to the level of abstraction: low (LA) vs. high (HA) (see Figure 7), performance in both levels are accompanied by an increase in the score. Notwithstanding, this was more pronounced in the case of low level of abstraction. This occurred in both the cognitive (left) and motor (right) tasks (performance scores in cognitive task, *HA*: 3.1 ± 26.6 vs. *LA*: 23.6 ± 31.5, *U* = 38, *p* < 0.05; performance scores in motor task, *HA*: 6.7 ± 16.6 vs. *LA*: 13.3 ± 11.4, *U* = 52, *p* = 0.08). As hypothesized, the effect of the level of abstraction during training was observed to induce behavioral and cognitive changes on the transfer of knowledge. The improvement of the scores in the trained task under high abstraction was lower than under low abstraction of the cognitive and motor tasks. However, when the complementary level of abstraction of the trained task was executed, higher scores are achieved by those who received the training under high abstraction in comparison to those who received the training under low abstraction. This suggests a better mobilization of knowledge under the high abstraction. In this sense, the transfer of knowledge as proxied by the exhibited skills is larger under the higher level of abstraction.

### 3.2. Analysis of mental workload

The results regarding the assessment of the mental workload contrast the change of workload that participants obtained in the first training session against the execution of the trained task (session 1 − Execution), comparing the type of training: virtual (VT) vs. real (RT); and level of abstraction: high (HA) vs. low (LA). The analysis was made for the cognitive and motor tasks.

#### 3.2.1. Assessment of the mental workload by type of training

**Figure 8:**
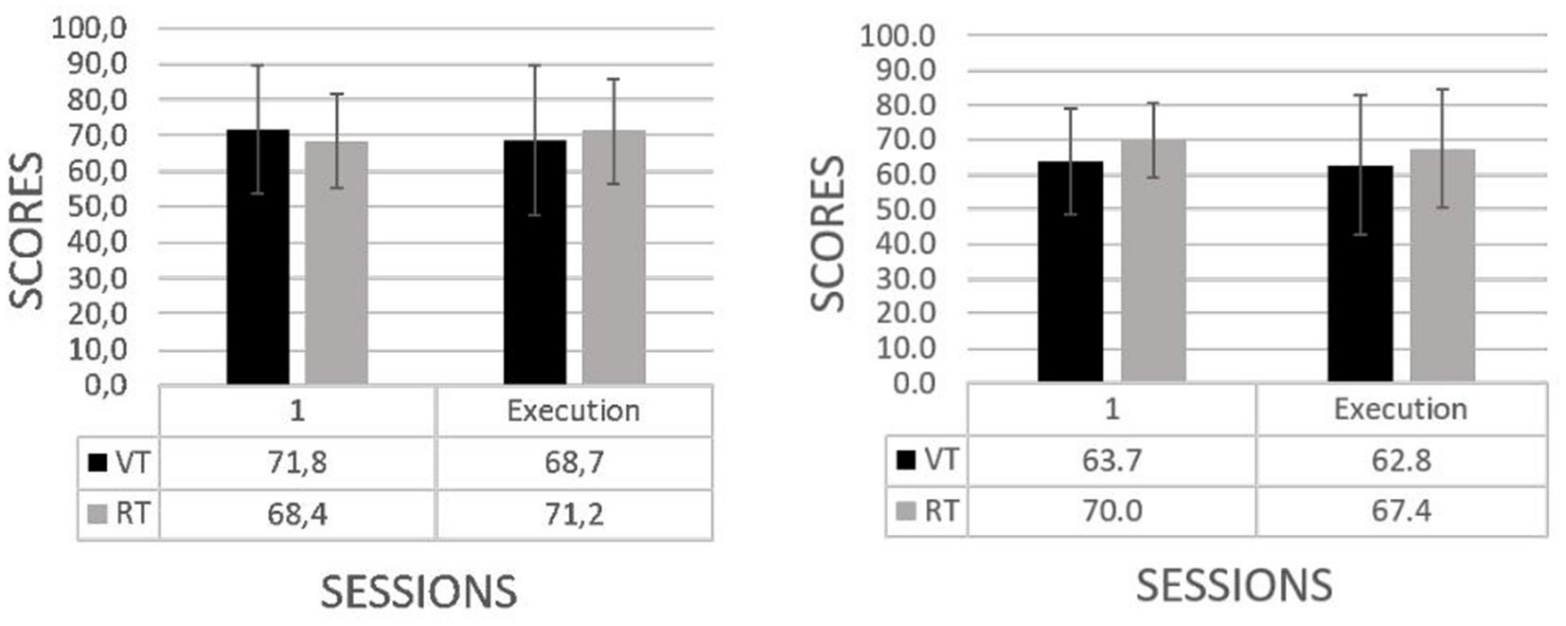
Mental workload (mean and standard deviations) in the first session of training and the execution of the trained task by type of training: virtual (VT) vs. real (RT) in the cognitive **(left)** and motor **(right)** tasks.

According to the type of training (see Figure 8), the group undergoing virtual training exhibited a moderate decrease in the use of cognitive resources by the end of the training of the cognitive (left) and motor (right) tasks (workload variability in the cognitive task, *VT*: −3.1 ± 6.0 vs. *RT*: 2.8 ± 12.5, *U* = 19.5, *p* < 0.05; workload variability in the motor task, *VT*: −0.9 ± 12.8 vs. *RT*: −2.6 ± 12.1, *U* = 67.5, *p* = 0.29). The principles of cognitive load theory indicate that cognitive load can arise from the following three major sources: intrinsic, extraneous, and germane (Chen et al., 2011). Our findings are in agreement with such sources. They show a more evident decrease of workload in the virtual vs. real training from the first session of training to the post-training. Such result is likely to reflect a better effective instructional virtual design against its real counterpart. This is supported by the intrinsic component of the cognitive load which is linked to the learning material.

#### 3.2.2. Assessment of the mental workload by level of abstraction

**Figure 9:**
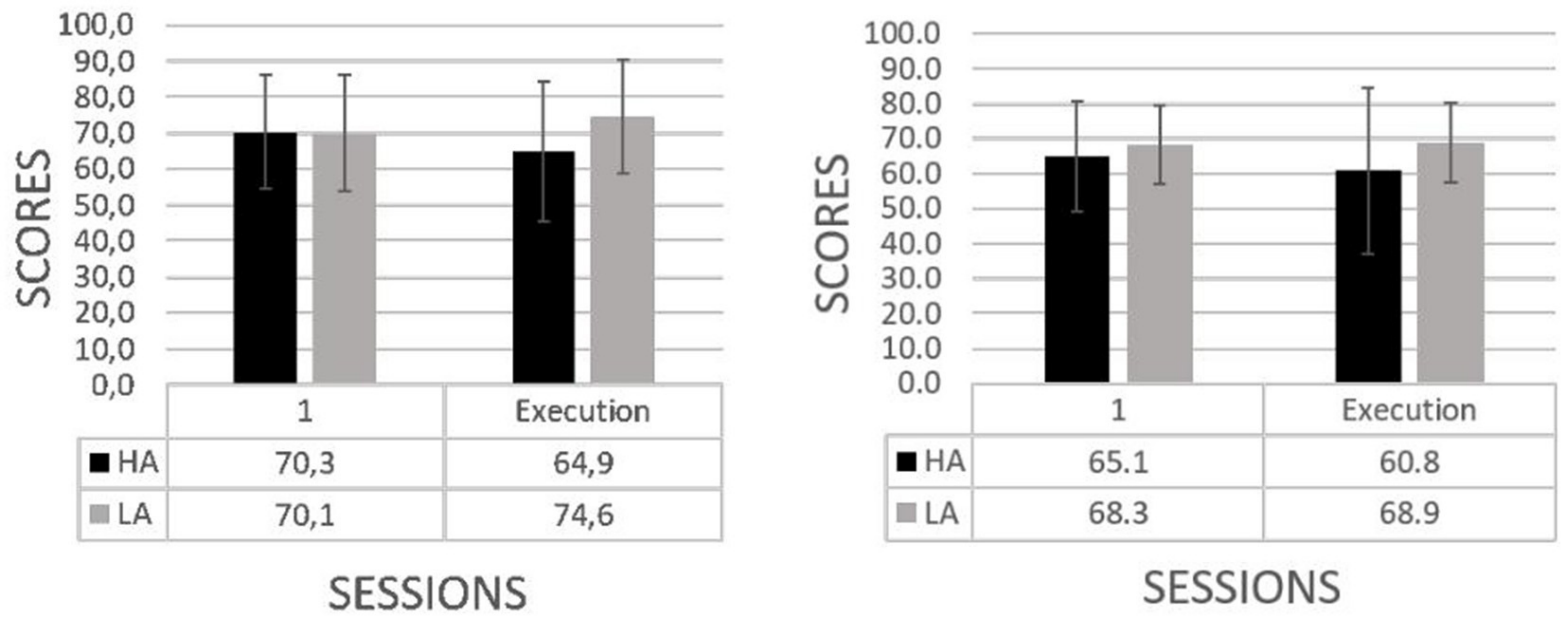
Mental workload (mean and standard deviations) in the first session of training and the execution of the trained task by abstraction level: high (HA) vs. low (LA) in the cognitive **(left)** and motor **(right)** tasks.

In the case of the level of abstraction (see Figure 9), the training with high abstraction was accompanied by decrements in the mental resources in the cognitive (left) and the motor (right) tasks. In contrast, training with low abstraction was accompanied by an increment in the mental workload resources at the end of the training period for the cognitive and motor tasks (workload variability in the cognitive task, *HA*: −5.4 ± 8.9 vs. *LA*: 4.5 ± 8.5, *U* = 12.5, *p* < 0.05; workload variability in the motor task, *HA*: −4.3 ± 16.1 vs. *LA*: 0.7 ± 7.1, *U* = 38.5, *p* < 0.05). Concerning to the high vs. low abstraction, our argument, supported by the cognitive load theory (Chen et al., 2011), points toward the germane component that suggests an increase of cognitive load over the investment of effort in the schema construction of knowledge which is accompanied with a decrease of the extraneous cognitive load during the process of construction. Altogether, this leads us to the hypothesis that the decrease of the mental resource in the high level of abstraction would be associated with the effective assimilation of the information.

### 3.3. Analysis of brain activity (EEG)

The longitudinal EEG recordings correspond to the learning process. As with other learning processes, it is expected that the brain activity becomes more efficient as knowledge becomes integrated at cortical level (Draganski et al., 2004). If this is the case, then this should be reflected in the segmented EEG recordings as a compression of the brain activity toward the earlier part of the recordings as the skill is integrated at brain level. Also, in line with the literature (Mestres-Missé et al., 2014), we expect higher activity associated to training under high abstraction as compared to training under low abstraction.

**Figure 10:**
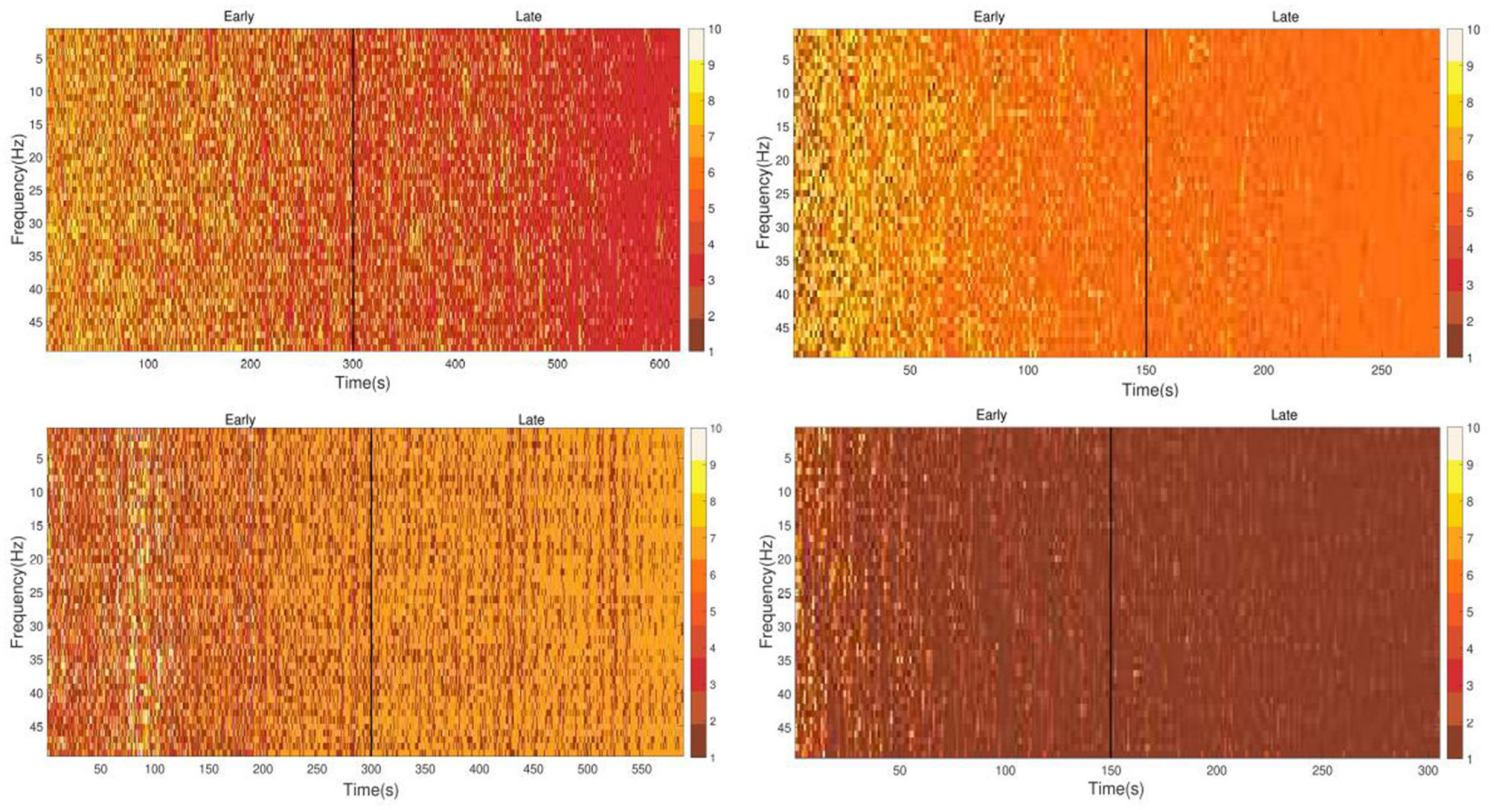
Comparison of representative patterns of TFT-epochs/cognitive subprocesses from physiological (EEG) measure and qualitative (mental workload) assessment. Top: subject trained with low level of task abstraction from VE. **(Left)** First session of training and **(right)** execution of trained task. In the mental workload assessment, the subject obtained the first session of training = 87.0 and when executing in RE the trained task = 84.3. Bottom: subject trained with high level of task abstraction from VE. **(left)** First session of training and **(right)** execution from RE of the trained task. In the mental workload assessment, the subject obtained the first session of training = 66.7 and when executing in RE the trained task = 65.3. Each color on the bar plots represents a cluster by HCA, which is assumed to represent a different cognitive subprocess.

Figure 10 shows the segmented TFT-epochs proxy of cognitive subprocesses corresponding to the first session of training (Figure 10, left) and the execution of the trained task (Figure 10, right) of the cognitive task in the VE under low (top) and high (bottom) levels of abstraction. Also, each segmentation was divided into early and late stages with the objective of analyzing the behavior of the brain during the same session. Segmentations have shown a distinctive pattern in the early stage of the 1st session of training and the executing of the trained task, the appearance of TFT epochs and ergo cognitive subprocesses in time and frequency were more notorious to compare with the late stage of the same segmentations.

Tables 2 and 3 summarize the entropy associated to the segmentations of activity patterns in the EEG of exemplary participants in their respective training schemes. Entropy was found to be smaller in late stage than the in early stage, both in the training and execution of the trained task. Regarding the level of abstraction, particularly, low abstraction with training from VE of the cognitive task increased its entropy from the early stage of the task of training to the early stage of the execution of the trained task and the same way with the late stage of both sessions. While high abstraction, in general, exhibited a decrease from the early stage of the task of training to the early stage of the execution of the trained task and the same way with the late stage of both sessions.

**Table 2:** Entropy of brain activity temporal segments in the *cognitive* task (a one-tailed Mann–Whitney U analysis was applied).

	**Low abstraction**			**High abstraction**		
	**Early**	**Late**		**Early**	**Late**	
**Virtual environment**						
1st. Day of training	1.04 ± 0.53	0.35 ± 0.29	*	1.04 ± 0.38	0.39 ± 0.23	***
Execution of trained task	1.07 ± 0.38	0.39 ± 0.27	**	0.61 ± 0.22	0.12 ± 0.10	**
**Real environment**						
1st. Day of training	1.45 ± 0.42	0.85 ± 0.39	*	0.93 ± 0.28	0.29 ± 0.21	*
Execution of trained task	1.09 ± 0.37	0.51 ± 0.38	*	0.82 ± 0.15	0.27 ± 0.24	**

*p ≤ 0.05, **p ≤ 0.01, and ***p ≤ 0.001.

**Table 3:** Entropy of brain activity temporal segments in the *motor* task (a one-tailed Mann–Whitney U analysis was applied).

	**Low abstraction**			**High abstraction**		
	**Early**	**Late**		**Early**	**Late**	
**Virtual environment**						
1st. Day of training	1.66 ± 0.17	0.85 ± 0.21	***	0.92 ± 0.43	0.42 ± 0.27	*
Execution of trained task	1.13 ± 0.39	0.40 ± 0.26	**	0.86 ± 0.46	0.29 ± 0.36	*
**Real environment**						
1st. Day of training	1.08 ± 0.59	0.54 ± 0.40	*p* = 0.11	1.43 ± 0.64	0.65 ± 0.50	*p* = 0.23
Execution of trained task	1.07 ± 0.61	0.52 ± 0.39	*p* = 0.07	1.05 ± 0.51	0.39 ± 0.51	*p* = 0.13

*p ≤ 0.05, **p ≤ 0.01, and ***p ≤ 0.001.

The decrease of TFT-epochs and consequently the associated cognitive subprocesses along the 1st session of training and the execution of the trained task is supported by entropy measurement that shows a decrease of values from the 1st day of training to the execution of the trained task which suggests, according to the literature, a better integration of knowledge.

## 4. Discussion

Virtual environments (VEs) have shown potential for modeling real environments (REs), presenting tasks in new ways during training and with the expectation of the transferring of the gained skills to the REs. However, not because they are technologically viable or thanks to their acceptance, it means they are or are not educationally relevant. The study of the performance and usefulness of VE in the task training remains an open research problem in fields such as human–computer interaction and cognitive neurosciences. There is still work to do to understand how the knowledge acquisition happens, at the behavioral and neurophysiological levels, and what task designs, mechanisms, or combinations of them facilitate the so-called transfer of knowledge from the VE training to the RE. Some mechanisms incorporated to VE, such as feedback, repetition, and motivation (Todorov et al., 1997; Rose et al., 2000; Bossard et al., 2008; Gupta et al., 2008; Girvan and Savage, 2019), have received thorough attention as they have been identified as critical for improving the user's performance in the training of tasks and their presence and emphasis in the training are expected to maximize the transfer of knowledge from VE. However, our knowledge of the transfer phenomenon still have severe limitations; among the most important one is the *adaptation* of acquired skills to tasks other than the one trained and of acquired knowledge to new environments and changing conditions in sit.

Several lines of research have investigated how abstract and concrete information is communicated within the cognitive system and processes that represent and generalize that information (Kiefer and Harpaintner, 2020). Literature shows that, compared to abstract concepts, concrete concepts are easier to investigate because their semantic content can be more clearly characterized (Barsalou and Wiemer-Hastings, 2005) and therefore, more quickly recognized, better remembered, and more resilient to brain damage (Binder et al., 2005). In contrast, abstract concepts have imposed challenges for all classes of theories because they are more complex, ambiguous, and apply to rather heterogeneous situations (Barsalou and Wiemer-Hastings, 2005; Hoffman et al., 2013). These views are also present in recent studies. Studies, such as Dove (2016) and Kiefer and Harpaintner (2020), have manifested the still-unresolved issues that lay underneath the representation of abstract concepts. Future research is encouraged to complement these results by investigating the dependency of abstract concept processing on different contexts, situational factors, task sets, habitual preferences, and differential attentional foci at hand, analogously to what has already been done in the case of concrete concepts (Popp et al., 2019; Kiefer and Harpaintner, 2020).

According to the previous idea, the influence of task abstraction presentation in training from VE on the transfer of knowledge and the mobilizing of knowledge toward other tasks has been evaluated here. Our hypothesis was that training with low level of abstraction would be associated with higher rates of transfer of knowledge in the trained task, but that would compromise on the ability to generalize the acquired knowledge to untrained tasks. In contrast, training under high level of abstraction would result in low rates of transfer of knowledge but shall be accompanied by greater ability to generalize to untrained tasks. Our interest in this research was motivated by the investigation of the influence of the abstraction presented in a task such training was carried out from a VE, in terms of studying the behavioral and neurophysiological effects on the transfer of knowledge and the generalization of knowledge toward other conditions of the training task. The results obtained from the scores and the physiological measurements have provided evidence for explaining the skills acquisition as well as the demand of brain resources associated with the phenomenon of the transfer of knowledge (knowledge construction from the training environment to the real environment). Increasing performance scores have shown that virtual training leads to reasonable mastering of the task. Furthermore, virtual training also leads to better post-training scores than real training. Likewise, according to the level of abstraction, the execution of the trained task under a low level of abstraction resulted in better performance scores than the execution under a high level of abstraction, further showing the same trend along the training sessions. However, participants trained using a higher level of abstraction achieved a better performance score when executing the task under the opposite level of abstraction just exactly as hypothesized. This evidenced that training under low abstraction helps to achieve a better performance in the training sessions as well as on the transfer of knowledge as long as the same concreteness was associated with the trained task. But importantly, the performance was compromised when attempting to reuse the knowledge when facing a higher abstraction of the trained task. This contrasts with the results achieved by those trained under a high level of abstraction. These results are supported by other studies that state that abstraction plays an important role in the representation of knowledge and its generalization. From the point of view of psychology, abstract concepts take longer to process lexical decision than concrete ones (Schwanenflugel, 2013), because the abstract referents cannot be experienced through sensation/perception (Nedjadrasul, 2017) and thus refer to a broad range of situations or constellations (Hoffman et al., 2013), and display a high degree of conceptual flexibility (Kiefer and Harpaintner, 2020). We believe that our results are in line with this literature. Associations experienced by participants using low abstraction allowed them to better apply what was learned in the analogous situation when challenged with the real scenario. However, these associations might have not been enough to mobilize the learning and thus the participants failed to recognize the complementary task and align the underlying structures toward other situations, i.e., failed to generalize. This, in contrast, was better achieved by the participants who trained under high abstraction who showed a better conceptual flexibility even though the mastering of the task was slower.

Regarding the mental workload experienced by the participants, post-training results indicated a slight workload reduction when participants were exposed to the high level of abstraction as well as virtual training. In turn, real training under the low level of abstraction exhibited, in general, a slightly higher cognitive workload. The workload ratings by the NASA-TLX have evidenced the experience of the participants facing out to the treatment, elucidating in the group trained with low abstraction a post-training mental overloaded factor which, according to the result of the performance score, is associated with a low task demand (Hart and Staveland, 1988), since participants were able to recognize and align easier the underlying structure of the low abstraction scheme and carry that construction toward its analogous task post-training in the real environment. On the contrary, the mastering of the task with the high level of abstraction was slower, presumably because of the heterogeneity of the abstract concepts (Harpaintner et al., 2020), resulting apparently in a workload mitigation by a continuous engagement to achieve the task (Hart and Staveland, 1988).

On the other hand, the segmented patterns in the EEG exhibited a bigger demand of brain resources at the beginning of training leading to reducted post-training, being more salient for the high abstraction of both the cognitive and motor tasks (see Tables 2, 3). This codification in the brain was assessed by the entropy quantified from the EEG signal, contrasting the early and late cortical responses during both the first training session as well as the post-training session. According to recent studies in neuroscience, this links to thermodynamic- and information-based models. Collell et al. (Collell and Fauquet, 2015) state that the measure of entropy is related to the energy expended within the brain and the organization of its information. That is, by encoding the new information in the brain, the energy expenditure is reduced in subsequent attempts on the same task. The activated neural paths could be more easily retrieved in future (Friston, 2010). Therefore, the patterns of neural activation segmented from EEG and quantified using entropy could suggest an association between the minimization of brain entropy post-training and the efficiency in adapting to task demands. It is likely that the increased efficiency in the use of brain resources does precede the improvement in behavioral performance (University, 2018). Notwithstanding, our experiment does not permit us to speculate further on this matter.

According to our results, the level of abstraction impacts the utilization of mental resources differently during tasks learning. While abstract structures (high abstraction) might be highly heterogeneous due to the semantic variability (Kiefer and Harpaintner, 2020), which in the present study was observed with a moderate acquisition of the knowledge during the training. Concrete structures (low abstraction), more likely to elicit a smaller set of possible associates (Welcome et al., 2011), showed a better training acquisition and transfer of knowledge to face the same concreteness to the trained task. Nevertheless, mobilizing of knowledge was improved with high abstraction training against low abstraction training where mobilization was easily affected due to mental representations acquired with this higher abstraction which allow to generalize elements to other circumstances. This evidence satisfies our departing hypothesis. Outlier values were traced back to issues in calibration of the task, particularly, the cognitive and motor tasks in real environment, which became less challenging for participants along the training as well as the recruitment was below the intended range, resulting in a small sample size.

The success of VE for training where real training is difficult remains subtly challenged by some apparently contradictory evidence regarding transfer of knowledge. We hypothesized that the level of task abstraction may influence the mobilization of knowledge and, therefore, explain such apparent contradiction.

## 5. Conclusion

Our findings in task performance have been consistent with performance scores and both estimates the brain resource consumption. The performance scores improved along the training and execution of the trained task. The segmentation cases showed higher variations of TFT-epochs/cognitive subprocesses in time and frequency, during the early stage, both initial and execution of training, but these variations decreased in the late stages. This result was supported by the decrease in mental workload score from the first day of training to the execution of the trained task. According to cognitive load theory (Chen et al., 2011), the phenomenon that occurs is linked to the brain becoming more efficient. Altogether, this evidence strongly supports our hypothesis, even though more experiments might be needed. Implications of this research may impact the way that VEs are developed for training purposes, as well as collaterally, impact the neuroscientific understanding of how we develop abstract skills.

There are several promising avenues for future research. From the point of view of human–computer interaction, with the definition of strategies to facilitate training conditions and enhance the potential transference of knowledge to adapt the acquired knowledge and skills as guidelines to perform and learn under changing situations or new environments. We ought to exploit the capabilities that virtual environments can provide through an interactive and more controlled experience while performing the training tasks. With the increasing availability of neuroimaging records, concomitant behavioral and brain activity monitoring facilitate observing how virtual features are impacting brain response and subsequently supporting performance. For instance, increasing the number of channels of EEG combined with source detection analysis (e.g., LORETA) would elucidate with finer spatiotemporal granularity aspects of processes of skills acquisition in a task under abstraction schemes. Finally, it would be interesting to systematically study and explain different patterns of neural activation and plasticity underlying processing of abstract schemes as a function of training or skills acquisitions.

## Data availability statement

The dataset used in this study will be made available by the authors, without undue reservation.

## Ethics statement

Ethical review and approval was not required for the study on human participants in accordance with the local legislation and institutional requirements. The patients/participants provided their written informed consent to participate in this study.

## Author contributions

VR, AC-Á, VR-M, and FO-E to conception and design of the work and drafted the work. VR, FO-E, and AM revised it critically for important intellectual content. VR, AC-Á, VR-M, NB-B, AM, and FO-E provide approval for publication of the content and agree to be accountable for all aspects of the work in ensuring that questions related to the accuracy or integrity of any part of the work are appropriately investigated and resolved. All authors contributed to the article and approved the submitted version.
